# Prognostic Implication of Intra-arterial 'Verapamil-Induced' Seizure in Patients With Aneurysmal Subarachnoid Hemorrhage: A Case Report

**DOI:** 10.7759/cureus.96883

**Published:** 2025-11-15

**Authors:** Narjis Alsaif, Buthaina ALobaid, Mazen ALotaibi, Fadhel ALmawlani, Ali Alahmed

**Affiliations:** 1 Critical Care Department, King Fahad Specialist Hospital, Dammam, SAU; 2 Neurosurgery Department, King Fahad Specialist Hospital, Dammam, SAU; 3 Interventional Radiology Department, King Fahad Specialist Hospital, Dammam, SAU

**Keywords:** aneurysmal subarachnoid hemorrhage, intra-arterial verapamil, neuro-critical care, neuro interventional, seizure

## Abstract

Aneurysmal subarachnoid hemorrhage aSAH is a medical emergency resulting from rupture of a cerebral aneurysm and bleeding into the potential space between the pia mater and arachnoid membrane. Complications include acute ischemia, brain edema, hydrocephalus, and increased intracranial pressure. Late complications include rebleeding, cerebral vasospasm, and systemic complications such as cardiac and pulmonary dysfunctions.

Cerebral vasospasm is a reversible and treatable cause of death after a ruptured aneurysm. Therefore, various strategies have been introduced as preventive and therapeutic options for cerebral vasospasm. One current option is intra-arterial verapamil administration, which rarely causes seizures. The prognostic implication of intra-arterial (IA) verapamil-induced seizure in patients with aneurysmal subarachnoid hemorrhage (aSAH) remains poorly understood.

We present a case of a young patient with aSAH who underwent successful endovascular management and coil embolization. The patient had complicated cerebral vasospasm and was treated with IA verapamil. During the intervention, he developed generalized tonic-clonic seizures, necessitating intubation and a prolonged stay in the intensive care unit (ICU). Notably, the patient achieved complete neurological recovery by the time of hospital discharge. This case suggests that the occurrence of seizures post-intra-arterial verapamil therapy in patients with aneurysmal subarachnoid hemorrhage is not necessarily associated with poor neurological outcome and might serve as a prognostic factor for reperfusion and, therefore, successful endovascular therapy.

## Introduction

Aneurysmal subarachnoid hemorrhage (aSAH) is a life-threatening neurological emergency associated with high prehospital mortality, ranging from 22% to 26% [1]. Additionally, the in-hospital mortality rate is approximately 19-20% globally [1]. Survivors often face long-term morbidity, limited functional status, and substantial economic burden. After rupture of the aneurysm, the blood escapes to the subarachnoid space, triggering subsequent cerebral events including increased intracranial pressure, activation of the inflammatory pathway, brain edema, and impairment of cerebral perfusion. This pathophysiology is thought to play a part in the mechanism of injury in aSAH. Additionally, narrowing of cerebral circulation, which is known as cerebral vasospasm (CV), and subsequent delayed cerebral ischemia (DCI) and infarction are other significant determinants that contribute to the high morbidity and mortality rates in these patients.

Prompt intervention, including early recognition and timely access to a neuro-interventional facility for early aneurysm securing via surgical clipping or endovascular coiling, and proactive management of emerging complications such as cerebral vasospasm and DCI, is crucial for improving patient survival and quality of life. Cerebral vasospasm is considered one of the major complications of aSAH. Treatment modalities for preventing and managing established VC include maintaining euvolemia, induced hypertension to enhance cerebral perfusion, and oral nimodipine. For an established CV, an aggressive approach is required, and endovascular techniques have been established for this purpose. Intra-arterial (IA) vasodilators, such as verapamil, have become a standard treatment for refractory vasospasm, either as adjunctive therapy to angioplasty or as sole therapy [1,2]. Although generally safe, IA verapamil has been rarely associated with seizures [3].In a retrospective cohort study of 188 consecutive patients presenting with aSAH, 86 patients developed symptomatic cerebral vasospasm and were subsequently treated with high-dose IA verapamil. Notably, 10 patients developed postprocedural seizures following IA verapamil therapy, all of whom were successfully managed with intravenous lorazepam [4].

The pathophysiology, clinical implications, and prognostic value of IA verapamil-induced seizure remain poorly understood. We present a case of aSAH in which IA verapamil induced seizure activity and the patient ultimately experienced a favorable outcome. This case potentially adds to the existing literature on the prognostic value of IA verapamil-induced seizure. We report this case to highlight the rarity of seizures as a complication of intra-arterial verapamil therapy. Interestingly, our patient achieved full neurological recovery and full functional independence, suggesting that seizures in this context do not necessarily associate with poor functional outcome.

## Case presentation

A 34-year-old male with a known history of hypertension on amlodipine 5 mg daily presented to the emergency department with a sudden-onset severe occipital headache, associated with two episodes of vomiting and a transient loss of consciousness lasting approximately five minutes. He denied any prior history of seizures, abnormal movements, or focal neurological symptoms. Notably, he reported experiencing two similar episodes of headache accompanied by dizziness during the week preceding admission to the hospital.

**Figure 1:**
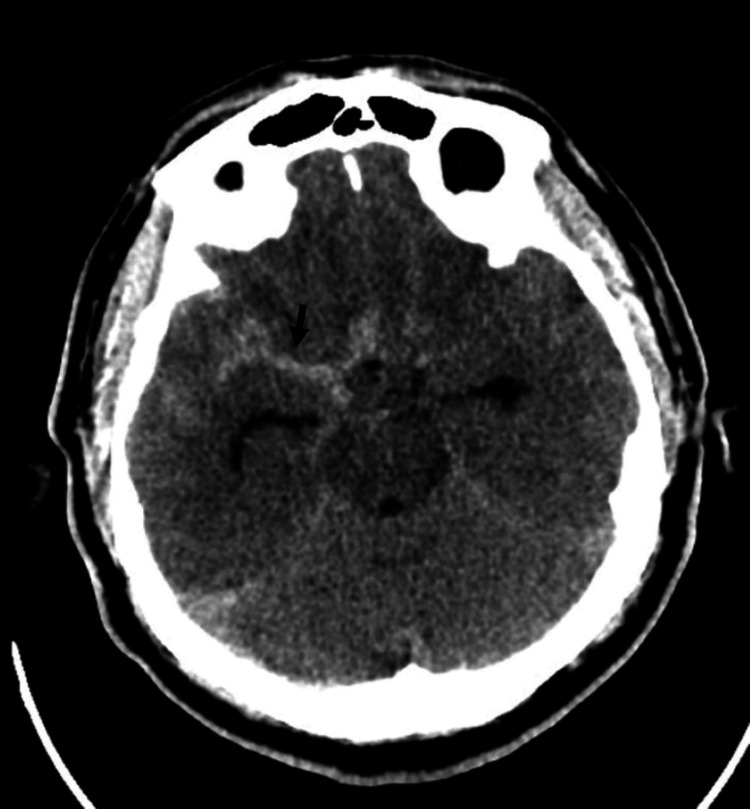
Computed tomography of brain without contrast (axial view) The image shows subarachnoid hemorrhage (black arrow) within the basal cisterns, more pronounced on the right side, right sylvian cistern, as well as the interhemispheric fissure and parasagittal sulci.

**Figure 2:**
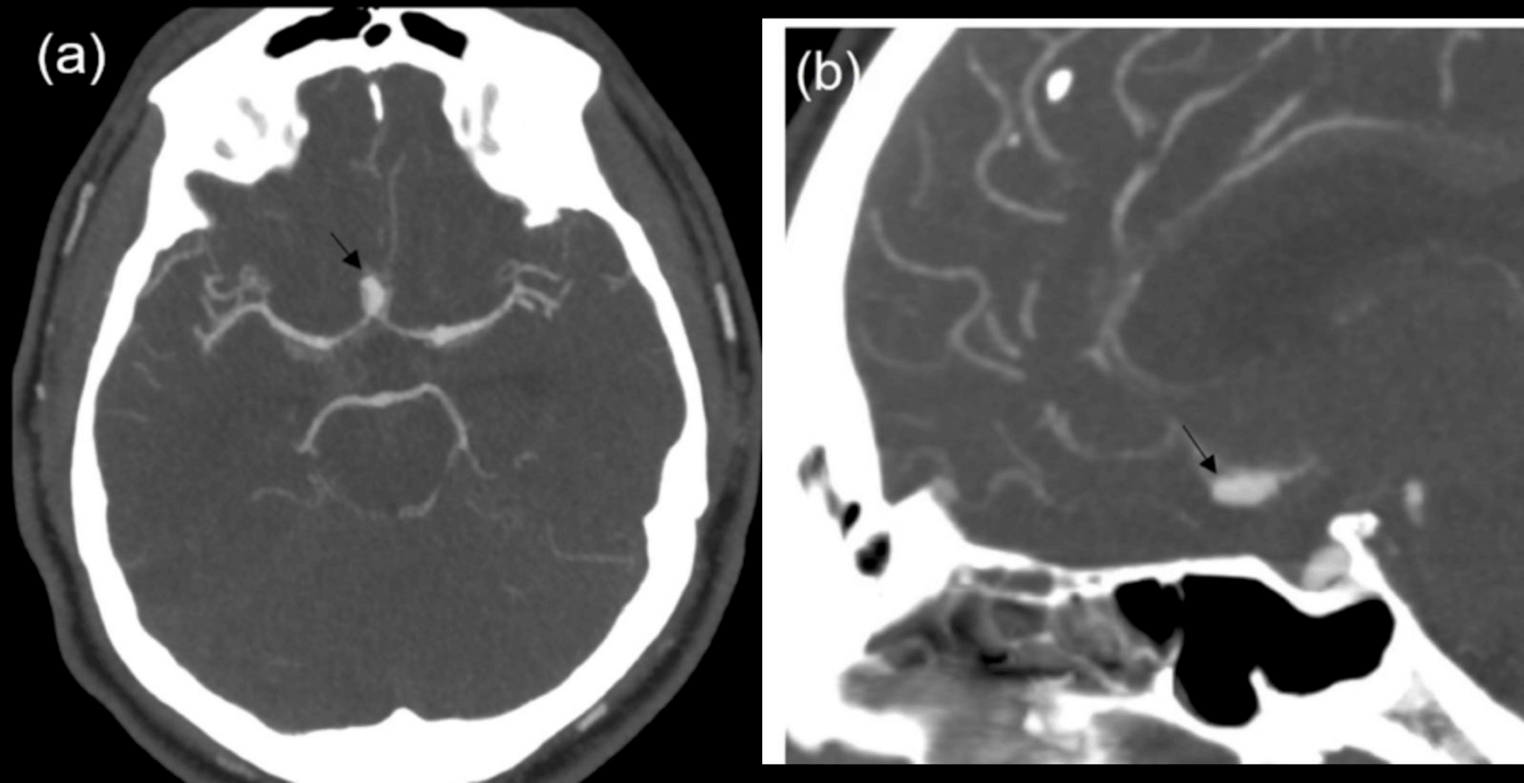
Computed tomography angiogram of brain Axial view (a) and sagittal view (b) show a saccular aneurysm directed anteriorly (black arrow) arising from the anterior communicating artery, which measures 0.9 x 0.6 cm.

On presentation, the patient was fully conscious, hemodynamically stable, alert, and oriented. Neurological examination revealed no cranial nerve deficits or motor/sensory abnormalities. Computed tomography (CT) of the brain revealed a subarachnoid hemorrhage (Figure 1). CT angiography demonstrated a 0.9 × 0.6 cm saccular aneurysm arising from the anterior communicating artery (ACOM), directed anteriorly (Figure 2), as well as a mild vasospasm of the supraclinoid segment of the internal carotid artery (ICA), proximal middle cerebral artery (MCA), and both anterior cerebral arteries (proximal and distal segments).

**Figure 3:**
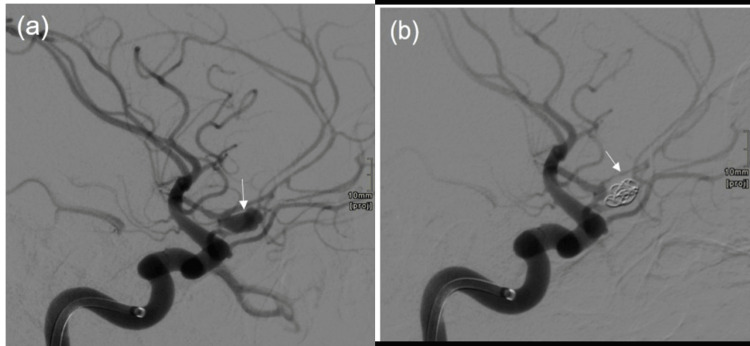
Angiography images (a)Pre-embolization left internal carotid artery showed the saccular anterior communicating artery (ACOM) aneurysm with associated small daughter sac arising from the dome (white arrow). The distal anterior cerebral artery (ACA) branches show vasospasm. (b) Post-embolization, the left internal carotid artery showed coiling of the ACOM aneurysm complicated by non-occlusive thrombus in the right A2 segment (white arrow).

The patient was admitted to the intensive care unit (ICU) and managed with intravenous fluids targeting euvolemia, blood pressure support with norepinephrine infusion (systolic blood pressure target of 100-140 mmHg), and nimodipine 60 mg orally every four hours. Within 24 hours, he underwent cerebral angiography and coil embolization of the aneurysm. During the procedure, thromboembolism was noted in the aneurysm and adjacent anterior cerebral artery (ACA) (Figure 3a), managed with two intra-arterial boluses of tirofiban 1 mg followed by infusion. Partial recanalization was achieved, and residual non-occlusive thrombus remained in the right A2 segment (Figure 3b). Vasospasm was noted, and nine milligrams of intra-arterial verapamil were administered. The patient remained neurologically stable and was returned to the ICU.

**Figure 4:**
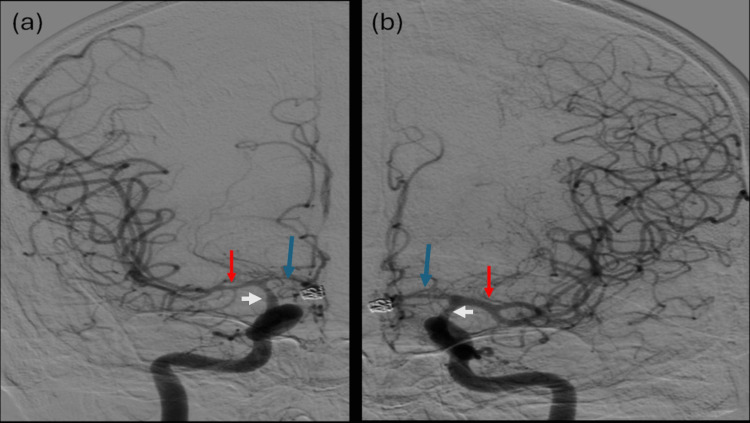
Cerebral angiography Angiography of the right (a) and left (b) obtained in the anterior-posterior projection shows the moderate vasospasm of the supraclinoid internal carotid artery (ICA) (white arrow) and proximal middle cerebral artery (MCA) (red arrow), more on the right side. The anterior cerebral artery (ACA)s (blue arrow) show moderate A1 and severe distal branches vasospasm bilaterally.

Twenty-four hours after initial presentation, the patient developed fluctuating left-sided weakness, with muscle power ranging from 0 to 3/5 in the upper limb (LUL) and 0 to 1/5 in the left lower limb (LLL). A non-contrast computed tomography (CT) scan of the brain revealed no new abnormality. The patient was subsequently transferred to the angiography suite, where cerebral angiography demonstrated bilateral vasospasm of the supraclinoid ICA, proximal MCA, and A1 segment of ACA, as well as severe vasospasm of the distal branches in the interhemispheric portion associated with sluggish flow (Figure 4). Intra-arterial verapamil injection, but after a total of 30 mg, the patient immediately experienced two episodes of tonic-clonic seizures. The seizures were promptly treated with intravenous midazolam (total dose: 10 mg), followed by intubation for airway protection. A repeat head CT scan showed no new intracranial pathology.

After stabilization, the patient was transferred to the ICU, where a physical examination showed a Glasgow Coma Scale score of 11/15 on mechanical ventilation and was able to obey simple commands. Muscle strength was 2/5 in the left upper limb (LUL) and 0/5 in the left lower limb (LLL). Laboratory tests, including blood glucose, calcium, sodium, and potassium, renal and liver function, were normal and excluded a metabolic cause of seizure. The patient was loaded with 2 g of levetiracetam and maintained 500 mg twice daily. Milrinone infusion increased to 1 mcg/kg/min along with hemodynamic support with vasopressor and fluid resuscitation as needed. 

Over the subsequent 72 hours, the patient demonstrated steady neurological improvement, with LUL power improving to 4/5, although LLL remained 0/5. The patient underwent two additional cerebral angiograms with intra-arterial verapamil injections (total dose 40 mg and 20 mg, respectively) into the bilateral internal carotid arteries due to persistent cerebral vasospasm. During these sessions, continuous monitoring and preparedness for potential seizure were ensured, and fortunately, no recurrence of seizure. Eventually, the patient was successfully extubated. By day 11 post-coiling, the patient demonstrated significant motor recovery in the left lower limb, improving to 4/5 in both LUL and LLL, and was transferred to the general ward for rehabilitation. By day 20, he was discharged home with complete functional recovery, fully independent and mild weakness on the left side (both upper and lower limb power 4/5) and instructed to continue on nimodipine (60 mg orally once daily) for a total duration of 21 days.

**Table 1:** Timeline table of clinical events ACOM: Anterior Communicating Artery, ICA: Internal Carotid Artery, MCA: Middle Cerebral Artery, ACA: Anterior Cerebral Artery, LLL: Left Lower Limb, LUL: Left Upper Limb, CV: Cerebral Vasospasm.

Relevant past medical history				
A 34-year-old male with a known history of hypertension on amlodipine 5 mg daily				
DAY	Clinical event	CT Angiography	Interventions	
Day1	Sudden-onset severe occipital headache, associated with two episodes of vomiting and a transient loss of consciousness lasting approximately five minutes	Subarachnoid hemorrhage saccular aneurysm arising from ACOM. Bilateral CV of the supraclinoid segment of the ICA, MCA and both ACA	ICU admission, Continuous monitoring, Euvolemia, Hemodynamic support and induced hypertension Nimodipine (60mg PO every 4 hours)	Coil embolization of the aneurysm, tirofiban injection for thromboembolism, IA verapamil (9mg) for the vasospasm
Day 2	Lest sided weakness (0/5 to 1/5 in LLL and 0/5 to 3/5 in LUL)	CV of bilateral supraclinoid ICA, MCA and ACA.		IA verapamil (30mg) injected, and the patient immediately experienced two episodes of seizures, The seizures were treated with midazolam, intubation, 2 grams of levetiracetam then 500 mg twice daily. Milrinone infusion increased to 1 mcg/kg/min
Day 3- Day 11	Steady neurological improvement (power LUL 4/5, LLL 0/5)	Two angiographies: persistent CV of bilateral ICA		In each angiographic session, IA verapamil injections (total dose 40 mg and 20 mg, respectively) into the bilateral ICA
Day 11- Day 20	Motor recovery (power 4/5 in both LUL and LLL)	No repeated images needed	Transferred to the general ward for rehabilitation.	
Day 20	Remain stable neurologically (both LUL and LLL power 4/5)	No repeated images needed	Discharged home, fully independent	

## Discussion

Individualized patient treatment strategies and a multidisciplinary approach are essential for managing patients with aneurysmal subarachnoid hemorrhage (aSAH). Collaboration between neurosurgeons, neuro-interventionists, intensivists, and dedicated rehabilitation specialists is crucial for improving survival and functional independence. Early detection and aggressive management of potential complications, including cerebral vasospasm (CV), are vital. CV may manifest as angiographic vasospasm (up to 70% of all aSAH cases), where patients remain asymptomatic, or as clinical vasospasm (up to 30% of patients with ruptured aneurysms), which can lead to permanent neurological deficits or even death. This is often precipitated by delayed cerebral ischemia (DCI) and possible infarction. Notably, the peak risk of CV occurs 6-10 days post-aneurysm rupture. This complication results from micro- or macro-narrowing, as well as diffuse or segmental narrowing, in the arterial cerebral circulation [1,3,4]. Preventive measures against CV include maintaining euvolemia, induced hypertension, and early initiation of nimodipine, a calcium channel blocker. Nimodipine is the only pharmacological agent recommended by the American Heart Association/American Stroke Association 2023 guideline [1]. Once CV is diagnosed, these interventions should be aggressively continued, and endovascular therapy should be initiated promptly. In recent years, various endovascular techniques, including mechanical and pharmacological approaches, have been introduced for managing established CV [1,4,5].

Intra-arterial vasodilator agents can achieve vasodilation of both proximal and distal cerebral vasculature branches. Although intra-arterial nimodipine and papaverine have been used historically, the availability of IA nimodipine is limited in many areas, and lA papaverine's use has been limited due to its neurotoxicity. lA verapamil, a calcium channel blocker, is widely available and has a favorable safety profile [6,7]. Common adverse effects include hypotension and bradycardia, while seizures have been reported rarely [3].

There is still uncertainty about the pathophysiology of verapamil-induced seizures, but it is hypothesized to involve cortical hyperexcitability secondary to reperfusion phenomena, particularly in ischemic areas [8]. The reperfusion theory is supported by a case series of three patients with severe stroke who experienced a seizure during tissue plasminogen activator (tPA) infusion. The repeated brain angiography ruled out any hemorrhage or other complications and demonstrated angiographic evidence of reperfusion of clotted arteries. All three cases had significant neurological improvement after reperfusion therapy, as well [4]. Our patient experienced two episodes of tonic-clonic seizures after receiving 30 mg of lA verapamil and subsequently achieved full neurological recovery. The only argument against the reperfusion theory in our case is the persistence of radiological vasospasm despite clinical improvement in patients’ neurological status. Alternatively, direct neurotoxicity of verapamil could be considered. However, the lack of repeated seizures with subsequent verapamil sessions suggests otherwise.

In a study by Ziu et al, four out of 36 patients diagnosed with aSAH and treated with IA verapamil for vasospasm experienced seizures. A correlation was found between verapamil dosage and seizure occurrence (median dose was 50 mg in the seizure group versus 35 mg in the non-seizure group) [9]. Notably, our patients had a seizure during the second angiography session after receiving 30 mg of IA verapamil, whereas the first session (9 mg of verapamil) was seizure-free, supporting the correlation between verapamil dose and seizure risk.

This case highlights a potential and serious complication of IA verapamil therapy: seizures occurring immediately after treatment, requiring prompt medical care and immediate action. Interestingly, seizures may indicate therapeutic reperfusion and a favorable functional outcome, rather than an adverse prognostic indicator. Finally, under close monitoring, seizure occurrence post IA verapamil does not preclude repeating therapy for recurrence vasospasm. Additionally, in a case series of total 86 patients with symptomatic vasospasm reported seizures in 10 patients post intra-arterial verapamil, all successfully treated with benzodiazepine without progression to refractory seizure or status epilepticus, similar to our case.

## Conclusions

This case illustrates a rare but important complication of intra-arterial verapamil administration in patients with aneurysmal subarachnoid hemorrhage, which is seizure, and highlights that such an event could be totally reversible, does not necessarily predict a poor outcome, and does not preclude the utilization of verapamil with persistent vasospasm. On the contrary, our patient demonstrated a complete functional recovery despite severe neurological compromise and intensive care needs. Further studies and case series are necessary to understand the true clinical implications of this phenomenon and to determine whether seizures in this context may serve as a marker of effective cerebral reperfusion.
